# Editorial: Neurobehavioural Mechanisms of Resilience and Vulnerability in Addictive Disorders

**DOI:** 10.3389/fnbeh.2020.644495

**Published:** 2021-01-20

**Authors:** Maria A. Aguilar, Nazzareno Cannella, Antonio Ferragud, Rainer Spanagel

**Affiliations:** ^1^Research Unit “Neurobehavioral Mechanisms and Endophenotypes of Addictive Behavior”, Department of Psychobiology, Faculty of Psychology, University of Valencia, Valencia, Spain; ^2^School of Pharmacy, University of Camerino, Camerino, Italy; ^3^Department of Psychology, University of Cambridge, Cambridge, United Kingdom; ^4^Institute of Psychopharmacology, Central Institute of Mental Health, Heidelberg University, Mannheim, Germany

**Keywords:** resilience, alcohol use disorder, substance use disorder, animal model, vulnerability

Substance use disorders (SUDs) and alcohol use disorder (AUD) entail serious global health, economic, and social problems. The efficacy of available addiction therapies is limited to subsets of responsive patients. A better understanding of the biological bases of transition from recreational drug use to drug abuse occurring in vulnerable drug users could help to tailor treatments and preventive interventions and would thus improve the quality of life of patients and consequently would reduce the negative impact of addiction on society. The aim of this Research Topic is to identify traits of resilience and vulnerability of SUDs and AUD and to unravel their neurobehavioral mechanisms. This Research Topic presents a collection of 16 contributions (three reviews, two min-review, nine original research papers, a brief research report, and one opinion paper) that provide evidence of critical individual-based differences modulating the response to different drugs of abuse (cocaine, alcohol, nicotine, opiates, and inhalants) and report preclinical findings on the efficacy of behavioral and pharmacological manipulations to prevent addiction-like behaviors.

The review by Kuhn et al. “*Understanding Addiction Using Animal Models*” discusses the advantages and disadvantages as well as the face validity of the most common preclinical models of addictive behavior and the attempts to better model individual vulnerability to drug abuse. These models have demonstrated the existence of individual genetic susceptibilities for traits such as sensation-seeking or impulsivity that contribute to vulnerability of addictive behavior.

Sex and age are two major variables that modulate the effects of drugs of abuse and the vulnerability of individuals to drug addiction. The review “*Sex Differences and the Role of Estradiol in Mesolimbic Reward Circuits and Vulnerability to Cocaine and Opiate Addiction*” by Kokane and Perrotti addresses the enhanced vulnerability of women to drug addiction and revises evidence that ovarian hormones are associated with faster progression into addiction. In female rodents, estradiol influences dopamine activity within the mesolimbic reward system that could explain why the rewarding effects of drugs and the response of animals to drug-associated cues are higher in females. The importance of age is highlighted by Guirado et al. in the mini-review “*A Critical Period for Prefrontal Network Configurations Underlying Psychiatric Disorders and Addiction*.” The authors analyze how early life and adolescence are critical periods in which exposure to drugs of abuse might lead to the formation of a particular prefrontal network configuration predisposing an individual to addition in adulthood. This network configuration is characterized by a predominance of inputs from the basolateral amygdala to the medial prefrontal cortex, the brain region responsible for higher cognitive functions and decision-making. A multiscale cerebral neurochemical connectome of the rat brain shows that the infra- and prelimbic cortices that comprise prefrontal cortex have very high out-going and in-going projections (Noori et al., 2017). This corresponds with the key role of prefrontal cortex in regulating cognitive functions. Traumatic events in early life and adolescence also contribute to shape a prefrontal network configuration predisposing to SUD. The review of María-Ríos and Morrow “*Mechanisms of Shared Vulnerability to Post-traumatic Stress Disorder and Substance Use Disorders*” focusses on this issue. SUD and post-traumatic stress disorder (PTSD) are often comorbid and could have a common etiology. Clinical and animal data suggest that some individuals have an intrinsic vulnerability that predisposes them to both PTSD and SUD. Dopaminergic, adrenocorticotropic, GABAergic, and glutamatergic neurobehavioral mechanisms, which underlie different emotional learning styles, may be involved in the etiological link between SUD and PTSD.

The opinion paper by Asensio et al. “*What Is the “Trigger” of Addiction?*” comments on the role of frustration in triggering a negative perception of the reality, an element shared in both, stress and addiction disorders. As relapse is frequently caused by intrapersonal determinants related to frustration, a dedicated therapeutic intervention could lead to increased relapse prevention.

The majority of original research papers in this Research Topic focuses on individual differences related with resilience or vulnerability to the effects of drugs of abuse in behavioral paradigms of preclinical research. The paper of Leite-Ferreira et al. “*Individual Differences in Hatching Time Predict Alcohol Response in Zebrafish*” reports that fishes classified as early or late emerging according to their egg emergence time showed different sensitivity to the effect of alcohol on locomotion and freezing. The work by Stafford et al. “*Individual Vulnerability to Stress Is Associated With Increased Demand for Intravenous Heroin Self-administration in Rats*” shows that rats with greater vulnerability to the behavioral and biological effects of an inescapable intermittent swim stress predicted the magnitude of individual demand of self-administered heroin weeks after the stress episode. In the same line, the research report by Calpe-López et al. “*Behavioral Traits Associated With Resilience to the Effects of Repeated Social Defeat on Cocaine-Induced Conditioned Place Preference in Mice*” identifies several behavioral traits that determine the long-term effects of social defeat stress on the rewarding properties of cocaine. Social defeat increased cocaine-conditioned place preference (CPP) but some mice are resilient to this effect of stress. In particular, mice showing less submission during defeat episodes, low novelty-seeking, high social interaction, and fewer depressive-like symptoms did not develop cocaine-induced CPP. Furthermore, the paper of Arenas et al. “*Prepulse Inhibition of the Startle Reflex as a Predictor of Vulnerability to Develop Locomotor Sensitization to Cocaine*” demonstrates that the baseline prepulse inhibition (PPI) levels of mice can predict their sensitivity to the locomotor effects of cocaine. Low-PPI mice presented low sensitivity to the motor effects of an acute dose of cocaine, but a higher increase of activity after repeated administration of the drug (behavioral sensitization) than High-PPI mice. Based on these and previous results - Low PPI presented a more persistent cocaine-induced place preference than High-PPI mice (Arenas et al., 2018) - it is suggested that a PPI deficit may be an endophenotype for cocaine use disorder. Finally, the research of Takahashi et al. “*Pavlovian to Instrumental Transfer Responses Do Not Correlate With Addiction-Like Behavior in Rats*” highlights the distinction between individual differences in the motivational impact of drug-associated cues and individual differences in the risk for addiction. Cocaine self-administration correlates with the level of Pavlovian-to-instrumental transfer (PIT), thus, a stronger PIT predicted improved learning of drug-cue association. However, in rats previously screened with the 0/3 criteria cocaine addiction model (Deroche-Gamonet et al., 2014), there are no differences in the PIT paradigm between addict-like and non-addict-like rats. This data suggests that stronger PIT may predict higher motivational impact of conditioned stimuli on drug self-administration and improved learning of drug-cue association rather than the risk to develop addiction as such.

Two research papers report on biological markers predicting the response to drug of abuse in humans. In the paper entitled “*Methylation Patterns of the HTR2A Associate With Relapse-Related Behaviors in Cocaine-Dependent Participants*” Land et al. show that the degree of methylation at several cytosine residues within the HTR2A promoter is positively correlated with impulsivity and attentional bias toward cocaine-associated cues in cocaine-dependent subjects. These results suggest that DNA methylation of the HTR2A gene may contribute to individual differences in behavioral effects that contribute to relapse. In the paper entitled, “*Default Mode Network Efficiency Is Correlated With Deficits in Inhibition in Adolescents With Inhalant Use Disorder*” Hernández-Álvarez et al. describe deficits in communication among brain regions involved in executive cognitive functions, mainly in the default mode network, of inhalant-consuming adolescents. These deficits may contribute to reduced inhibitory control and sequential planning seen with chronic inhalant abuse.

As commented before, animal models of drug addiction contribute to the development of therapies for SUD. Four papers in this Research Topic focus on the potential pharmacological and behavioral strategies to treat addiction to several drugs of abuse. In the paper “*Dopamine D3 Receptor Antagonism Reverses the Escalation of Oxycodone Self-administration and Decreases Withdrawal-Induced Hyperalgesia and Irritability-Like Behavior in Oxycodone-Dependent Heterogeneous Stock Rats*” de Guglielmo et al. report that the D3 dopamine antagonist VK4-116 is effective to prevent the motivation for oxycodone in a model of extended access and the negative emotional states associated with its withdrawal. The brief report by Garcia-Rivas et al., “*Varenicline Targets the Reinforcing-Enhancing Effect of Nicotine on Its Associated Salient Cue During Nicotine Self-administration in the Rat*” emphasized the individual differences in the capacity of varenicline to reduce the enhancement induced by nicotine in the rewarding properties of salient environmental stimuli associated with this drug. These results suggest that varenicline might be more beneficial in those smokers who are more sensitive to environmental stimuli associated with nicotine consumption. The original research of Khoo et al. “*Comparing ABA, AAB, and ABC Renewal of Appetitive Pavlovian Conditioned Responding in Alcohol- and Sucrose-Trained Male Rats*” also addresses the importance of drug-associated cues in the treatment of addiction. From a treatment perspective, the data of this study suggests that exposure-based treatments for SUDs might benefit from implementation in real-world, drug-use contexts. Finally, the mini-review by Kuijer et al. “*Retrieval-Extinction and Relapse Prevention: Rewriting Maladaptive Drug Memories*?” focuses on the application memory reconsolidation to disrupt maladaptive drug-memories that trigger relapse. After retrieval, maladaptive drug memories are destabilized (by an amnestic agent or by an extinction training) and reconsolidated in an updated form. It is suggested that retrieval-extinction protocols may have promising applications to help preventing relapse to drug-seeking and individual-based differences may influence the therapeutic outcomes.

We as Research Topic Editors are grateful for excellent contributions and are convinced that the here presented collection of papers summarize critical factors that contribute to a better understanding of the neurobehavioral mechanisms underlying resilience and vulnerability to addictive disorders.

## Author Contributions

All authors listed have made a substantial, direct and intellectual contribution to the work, and approved it for publication.

## Conflict of Interest

The authors declare that the research was conducted in the absence of any commercial or financial relationships that could be construed as a potential conflict of interest.
